# Nicotinic Acetylcholine Receptor Subunits α4 and α5 Associated with Smoking Behaviour and Lung Cancer Are Regulated by Upstream Open Reading Frames

**DOI:** 10.1371/journal.pone.0066157

**Published:** 2013-07-02

**Authors:** Marlene Eggert, Eric Aichinger, Michael W. Pfaffl, Ortrud K. Steinlein, Martina Pfob

**Affiliations:** 1 Institute of Human Genetics, University Hospital, Ludwig-Maximilians-University Munich, Munich, Germany; 2 Physiology Weihenstephan, Center of Life and Food Sciences Weihenstephan, Technical University Munich, Freising-Weihenstephan, Germany; Neuroscience Campus Amsterdam, VU University, The Netherlands

## Abstract

Nicotinic acetylcholine receptor subunits (nAChR) are associated with different aspects of smoking behaviour as well as with smoking related disorders. Several of these subunits have been found to be upregulated in smokers or differentially expressed in lung tumor cells. The mechanisms behind these observations are not known but assumed to be mainly post-transcriptional. Many post-transcriptional mechanisms are initiated by functionally relevant sequence motifs within untranslated gene regions, such as upstream open reading frames (uORFs). We performed a systematic search in all smoking-associated neuronal nAChR subunits and identified functionally relevant uORFs in *CHRNA4* and *CHRNA5*. Luciferase experiments showed that these uORFs are able to significantly decrease protein expression. Our quantitative real-time PCR (qPCR) results strongly suggest that the observed effects originate at the translation rather than at the transcription level. Interestingly, the *CHRNA4* uORF was only functionally relevant when expressed in the shorter isoform of this gene. Therefore, the data presented in this study strongly points towards an important role of uORFs within the 5′UTR of *CHRNA4*-isoform 1 and *CHRNA5* as regulators of protein translation. Moreover, the shared uORF of *CHRNA4*-isoform 1/isoform 2 represents the first example of a sequence context-dependent uORF.

## Introduction

Cigarette smoking is known as a major risk factor for cardiovascular disease and smoking-associated malignancies like lung cancer [1]. Yet, approximately every third adult worldwide smokes and the number is increasing [2]. Therefore, it is not surprising that great efforts are made to uncover the causes of nicotine dependence, as well as to develop new strategies for prevention and therapy. Nicotine acts as an acetylcholine agonist that is able to bind to neuronal nicotinic acetylcholine receptors (nAChR). The nAChR form heterogeneous and homogeneous pentameric ion channels with a diverse expression pattern in neuronal and non-neuronal tissues [3]. Consequently, many of the genes coding for nAChR subunits are suspected to play a key role regarding nicotine dependence. Several nAChR subunit genes have already been linked to smoking and smoking-related diseases. For example, *CHRNA7*, the gene coding the α7 nAChR subunit was shown to repress airway basal cell proliferation and to remodel the lung epithelium. In addition, the hypothesis emerged that dysregulated α7 expression gives rise to preneoplastic transformations [4], [5]. Genetic variants within the coding region, but also in the promoter region of *CHRNA4* are associated with both subjective responses to smoking and smoking cessation outcomes [6]–[10]. However, most studies that have been published on the topic of cholinergic receptors and nicotine dependence/smoking related diseases point towards the nAChR gene cluster on chromosome 15. The polymorphisms within this region that are most consistently reported to be associated with smoking quantity, nicotine dependence, lung cancer and peripheral arterial disease are located either in the *CHRNA5* or *CHRNA3* gene [11]–[16]. Additionally, associations between *CHRNB3* polymorphisms and response to first time tobacco use were described [17].

The mechanisms by which nAChR genes associated with nicotine dependence are regulated are not known in detail so far. In general, gene expression can be modified either pre- or post-transcriptionally. The latter mechanisms are often mediated by cis-acting sequence motifs localized within the untranslated regions (UTR) that are present up- and downstream of most eukaryotic gene coding regions. Several motifs in the 5′UTR responsible for translational regulation have been identified in eukaryotic genes such as internal ribosome entry sites, iron responsive elements and upstream open reading frames (uORFs). The latter elements can play a crucial role in the regulation of gene expression. A growing body of evidence shows that uORFs are able to affect protein translation in various ways. Scanning ribosomes translating a uORF might not be able to reinitiate further downstream at the coding region ATG, or might stall at the uORF, blocking other ribosomes (ribosomal roadblock). Alternatively, the stop codon of the uORF might be mistaken as a nonsense mutation, triggering nonsense mediated decay. [18], [19]. Furthermore, the uORF protein itself might modulate translation, as indicated by mutagenesis experiments introducing missense mutation within uORF sequences [20]. All these mechanisms are likely to increase the functional variability of genes within populations. The functional importance of uORFs is further emphasized by the finding that mutations within them are able to cause human diseases such as hereditary thrombocythaemia or Marie Unna hereditary hair loss [21], [22].

In the present study we performed a systematic search for functionally relevant uORFs in nAChR subunits that are suspected to be involved in nicotine dependence and smoking-related diseases. Our experiments revealed that some uORFs contribute to the regulation of nAChR protein expression.

## Materials and Methods

### *In silico* analysis of putative uORFs in the 5′UTR of α3, α4, α5, α7, and ß3 nAChR subunits

**Figure 1:**
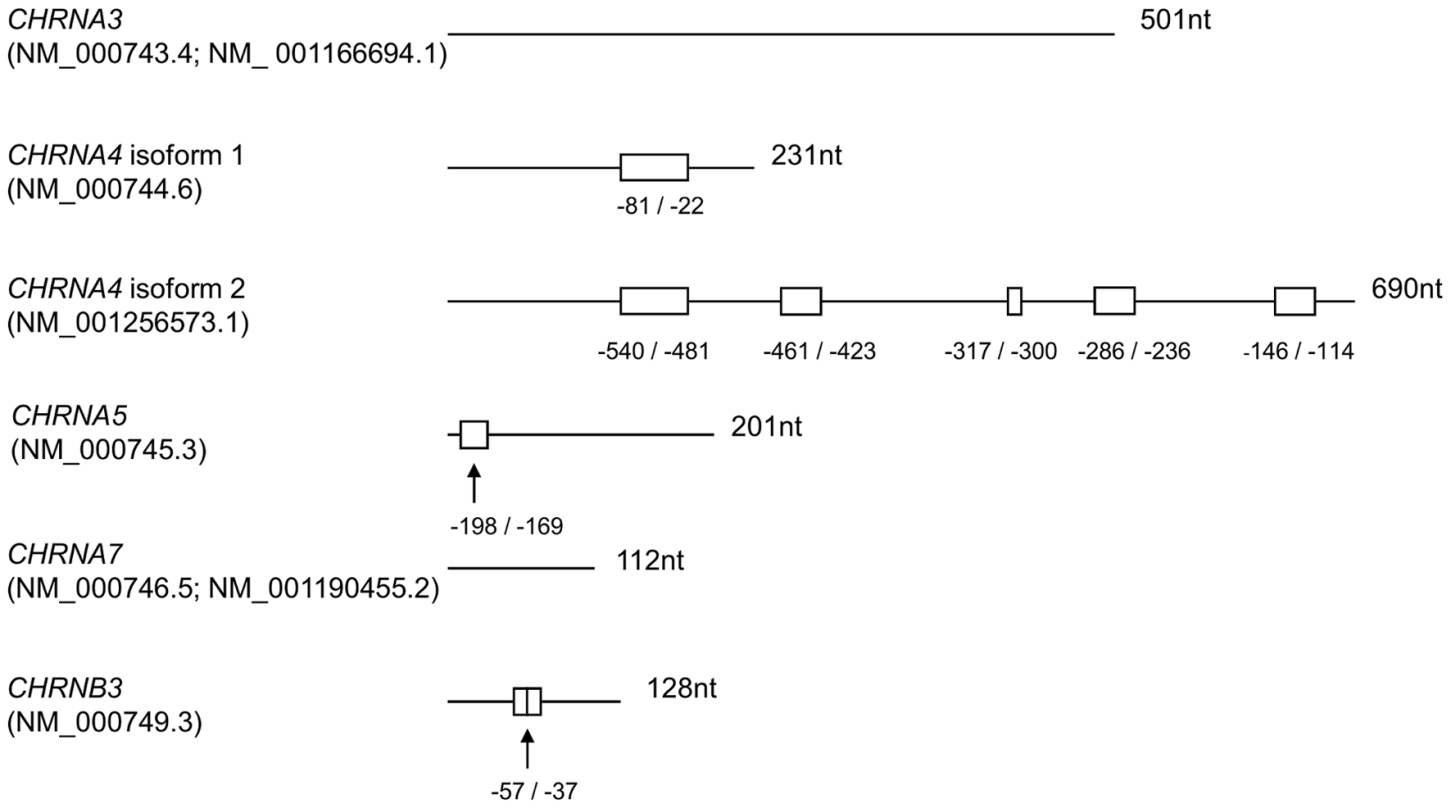
5 ′**UTRs of nicotine dependence-associated nAChR subunit genes with putative uORFs.** The bp position for each uORF (start/stop) is depicted starting from the main start codon in 5′- direction. *CHRNA4* isoform 1 and isoform 2 differ in their coding region, with a further downstream translation initiation for isoform 2, that elongates its 5′UTR. *CHRNA4* isoform 2 was not published until 2012 and has not been functionally analysed before. Squares, uORFs; arrows, SNPs

5′UTRs of *CHRNA3, CHRNA4* isoform 1 and 2, *CHRNA5, CHRNA7* and *CHRNB3* (coding for nAChR subunits α3, α4, α5, α7 and ß3) were screened for in-frame start and stop codons upstream of the main start codon with the StarORF Finder (http://star.mit.edu/orf/runapp.html). SNPs validated by SNP gene view of NCBI and located within the uORF sequence or creating/deleting a uORF were considered for investigation (Fig. 1).

### Plasmid construction

Firefly luciferase vector pGL4.10 (AY738222.1) and renilla luciferase vector pGL4.74 (AY738230.1) were purchased from Promega (Mannheim, Germany). The TK-Promoter sequence was cut out of pGL4.74 with KpnI and XhoI (Fermentas, St. Leon-Rot, Germany) after generating a XhoI restriction site at position bp 783 using geneart site-directed mutagenesis system (Invitrogen, Karlsruhe, Germany). After KpnI and XhoI digestion of pGL4.10, the TK-Promoter sequence was ligated into the multiple cloning site of pGL4.10 upstream of the firefly luciferase coding sequence. The 5′UTR inserts of the nAchR subunits α4 isoform 1 and 2, α5 and ß3 were synthesized (MWG Eurofins, Ebersberg, Germany) and cloned into pGL4.10 with XhoI and NcoI (Fermentas, St. Leon-Rot, Germany) directly between the TK-Promoter and the firefly luciferase coding sequence. After cloning the inserts were confirmed by sequencing. To create constructs lacking a certain uORF, the ATG of the uORF was mutated to TTG. Likewise, SNP alleles within uORFs were exchanged by site-directed mutagenesis. For *CHRNA4* isoform 1 (NM_000744.6), harbouring one putative uORF, two constructs were created: one with the wildtype mRNA (*CHRNA4*-iso1-1) and one with the mutated uORF start codon (*CHRNA4*-iso1-2). For a subsequent test (see results), two more constructs were created: one in which the stop codon of the uORF was mutated (TAG to TAC), but the firefly start codon stayed intact (*CHRNA4*-iso1-3), and one lacking both the stop codon of the uORF and the firefly start codon (ATG to TTG) (*CHRNA4-*iso1-4).

For *CHRNA4* isoform 2 (NM_001256573.1), possessing five putative uORFs, six constructs were created: one with the wild type mRNA (*CHRNA4*-iso2-uORF1-5) and five constructs in which each one of the five uORFs was switched off, numbered accordingly, starting with the most 5′ located uORF (*CHRNA4*-iso2-uORF1; *CHRNA4*-iso2-uORF2; *CHRNA4*-iso2-uORF3; *CHRNA4*-iso2-uORF4; *CHRNA4*-iso2-uORF5).

For *CHRNA5* (NM_000745.3), harbouring one putative uORF containing one SNP, four constructs were created: one with the allele guanine and intact/switched-off uORF (*CHRNA5*-1; *CHRNA5*-2) and one with the allele adenine and intact/switched-off uORF (*CHRNA5*-3; *CHRNA5*-4). For a subsequent test (see results), two more constructs were created: one in which the stop codon of the uORF was mutated (TAG to TAC), but the firefly start codon stayed intact (*CHRNA5*-5), and one lacking both the stop codon of the uORF and the firefly start codon (ATG to TTG) (*CHRNA*56).

For *CHRNB3* (NM_000749.3), harbouring a putative uORF and a SNP with the major allele adenine creating a uORF start codon, three constructs were used: one with intact uORF start codon generated by the SNP allele adenine (*CHRNB3*-1); one with the minor SNP allele guanine deleting the first uORF start codon (*CHRNB3*-2) thus leading to a truncated uORF and a third construct with the SNP allele guanine in which the start codon of the truncated uORF is switched off (*CHRNB3*-3). An overview of the constructs used in our experiments is shown in Table 1.

**Table 1:** List of plasmid constructs for luciferase assay and qPCR.

Gene	Clone	uORF			1CDS
		ATG	SNP	Stop	ATG
***CHRNA4***					
isoform 1	*CHRNA4*-iso1-1	+		+	+
	*CHRNA4*-iso1-2	−		+	+
	*CHRNA4*-iso1-3	+		−	+
	*CHRNA4*-iso1-4	+		−	−
isoform 2	*CHRNA4*-iso2-uORF1-5	+		+	+
	*CHRNA4*-iso2-uORF1	−		+	+
	*CHRNA4*-iso2-uORF2	−		+	+
	*CHRNA4*-iso2-uORF3	−		+	+
	*CHRNA4*-iso2-uORF4	−		+	+
	*CHRNA4*-iso2-uORF5	−		+	+
***CHRNA5***	*CHRNA5*-1	+	^2^g	+	+
	*CHRNA5*-2	−	^2^g	+	+
	*CHRNA5*-3	+	^3^a	+	+
	*CHRNA5*-4	−	^3^a	+	+
	*CHRNA5*-5	+	^2^g	−	+
	*CHRNA5*-6	+	^2^g	−	−
		ATG	ATG	Stop	ATG
***CHRNB3***	*CHRNB3*-1	+	+	+	+
	*CHRNB3*-2	−	+	+	+
	*CHRNB3*-3	−	−	+	+

1 coding sequence; ^2^guanine; ^3^adenine; +, present; −, non-functional

### Cell culture

The human embryonic kidney cells (HEK) 293 were purchased from Cell lines service (CLS, Eppelheim, Germany). Culturing of the cells was performed in T75 flasks in monolayer with DMEM (Sigma-Aldrich, Hamburg, Germany) containing 4.5 g glucose, 10% FBS, 1% L-Glutamine and 1% Penicillin/Streptomycin (Invitrogen, Karlsruhe, Germany). The cells were maintained in a humidified atmosphere at 37°C and 5% CO_2_.

HEK 293 cells were co-transfected with the reporter plasmid pGl4.10+TK containing the 5′UTR of α4 isoform 1 and 2, α5 and ß3, respectively, and the control plasmid pGL4.74 at a ratio 20∶1 using TransIT ®-LT1 Transfection Reagent (MoBiTec, Göttingen, Germany) with 24 h transfection prior to luciferase assay and qPCR, respectively.

### Luciferase assay

HEK293 cells were seeded 24 h prior to transfection with 3×10^5^ cells in 24 well plates (Greiner bio-one, Frickenhausen, Germany). The firefly and renilla luciferase activities were measured with the TRiStar LB941 (Berthold Technologies, Bad Wildbad, Germany) by the Dual-Glow® luciferase assay system (Promega, Mannheim, Germany) according to the manufacturer's protocol. The ratio of firefly luciferase and renilla luciferase activity was determined and normalized to pGL4.10+TK. The various 5′UTR constructs are expressed as fold change to pGL4.10+TK.

All experiments were repeated independently three times with triplicate samples. A two-tailed t-test was used to compare the values of the test samples and the control samples. A *p* value of *p*<0.05 was considered statistically significant. All data are expressed as fold change and as mean ± SEM.

### qPCR

Quantitative real-time PCR was performed if luciferase assay showed significant results. Co-transfection with the various constructs was performed as described above. RNA was extracted by using a Qiagen RNAeasy kit, including DNase treatment of 10 min, according to the manufacturer's protocol (Qiagen, Hilden, Germany). First-strand cDNA synthesis was carried out using 1 µg of total RNA from each transfection as starting material (iScript™ cDNA synthesis kit, Bio-Rad, Munich, Germany). Real-time PCR was performed targeting firefly luciferase (target) and renilla luciferase (control) coding sequence using the following primers firefly-fwd 5′- TGCAACACCCCAACATCTTC-3′ and firefly-rev 5′- CCTTTAGGCACCTCGTCCAC-3′; renilla-fwd 5′- AATGGCTCATATCGCCTCCT-3′ and renilla-rev 5′-CACGACACTCTCAGCATGGA-3′. Amplification efficiency and test linearity (correlation coefficient R^2^) were assessed for each primer pair (Fig. S1). The reactions were carried out in 20 µl volumes containing 10 µl SsoFast^TM^ EvaGreen^®^ Supermix (Bio-Rad, Munich, Germany), 125 nM of each primer, 7 µl molecular biology grade water and 1 µl of each template after cDNA synthesis. Thermal cycling consisted of denaturation (98°C for 5 s), annealing and extension (60°C for renilla; 62°C for firefly for 5 s), performed in 50 cycle steps in the Mini Opticon CFD-3120 cycler (Bio-Rad, Munich, Germany). Melting curve analysis ranged from 65°C to 95°C with 0.5°C intervals. All experiments were repeated independently three times with triplicate technical samples. A two-tailed t-test was used to compare the values of the target samples and the control samples. A *p* value of *p*<0.05 was considered statistically significant.

## Results

### *CHRNA3* and *CHRNA7*

*In silico* analysis showed that neither the 5′UTR of *CHRNA3* isoform 1(NM_000743.4) and isoform 2 (NM_ 001166694.1) nor of *CHRNA7* isoform 1 (NM_000746.5) and isoform 2 (NM_001190455.2) contain putative uORFs (Fig. 1). Therefore, these two subtype genes were not included in the study.

### *CHRNA4* isoform 1 5′UTR harbours a functional uORF

**Figure 2:**
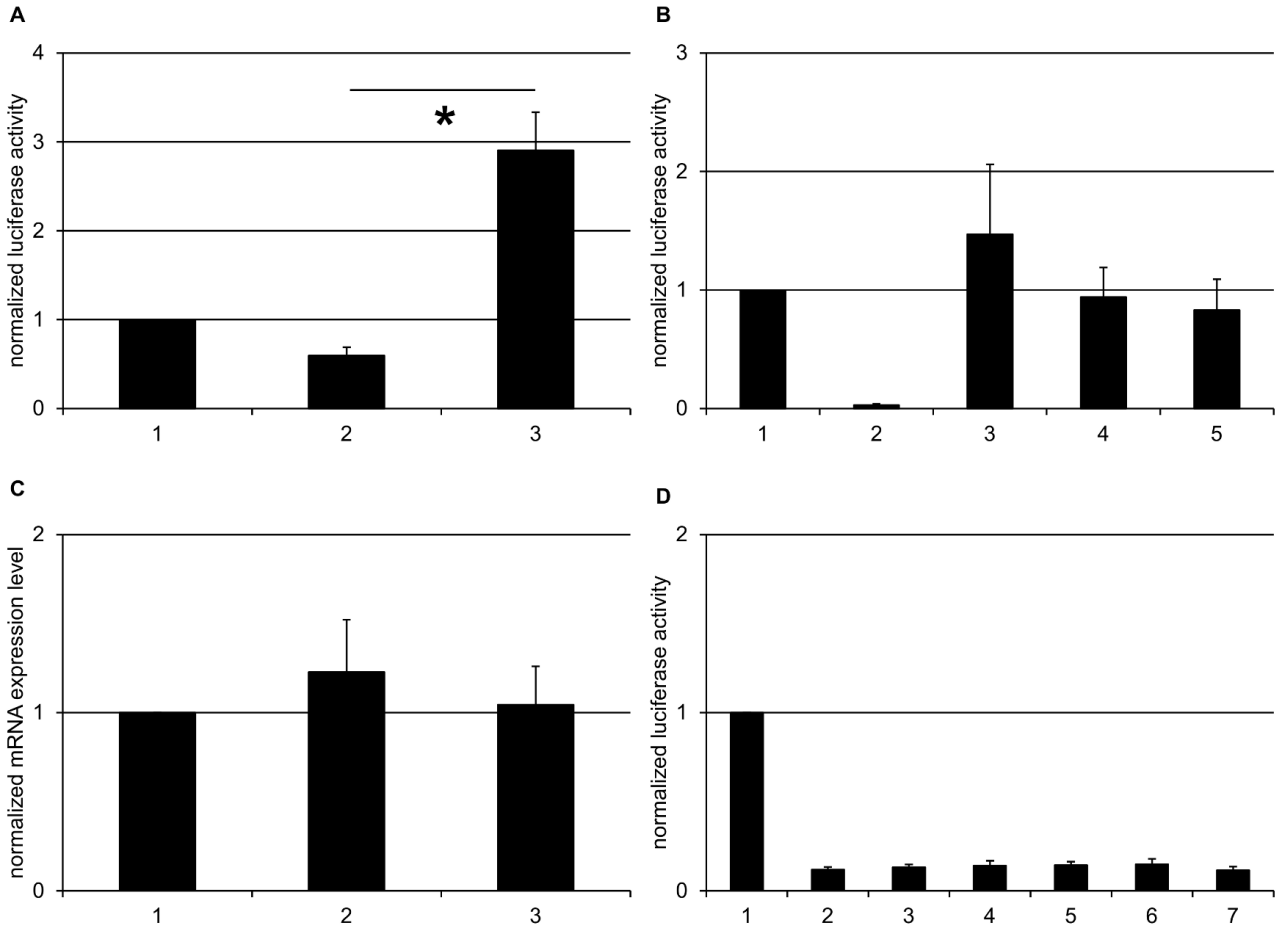
*CHRNA4* isoform 1 contains a post-transcriptionally functional uORF, but not *CHRNA4* isoform 2. **A:** Luciferase assay of *CHRNA4* isoform 1 5′UTR resulted in significant increase in protein expression when switching off the uORF. Fold change of firefly activity normalized to pGl4.10 +TK, is illustrated for the three different constructs; 1, pGl4.10+TK; 2, *CHRNA4*-iso1-1; 3, *CHRNA4*-iso1-2. **B:** The uORF ATG of *CHRNA4*-iso1 is able to initiate translation, resulting in an elongated firefly protein. Fold change of firefly activity normalized to pGl4.10 +TK, is illustrated for the five different constructs; 1, pGl4.10+TK; 2, pGl4.10; 3, *CHRNA4*-iso1-2; 4, *CHRNA4*-iso1-3; 5, *CHRNA4*-iso1-4. **C:** Relative qPCR for *CHRNA4-iso1* 5′UTR showed no significant differences of mRNA amount when comparing intact with deleted uORF. Fold change of firefly mRNA normalized to pGl4.10 +TK, is illustrated for the three different constructs; 1, pGl4.10+TK; 2, *CHRNA4*-iso1-1; 3, *CHRNA4*-iso1-2. **D:** None of the five uORFs of *CHRNA4*-iso2 5′UTR showed significant results. Fold change of firefly activity normalized to pGl4.10 +TK, is illustrated for the seven different constructs; 1, pGl4.10+TK; 2, *CHRNA4*-iso2-uORF1-5; 3, *CHRNA4*-iso2-uORF1; 4, *CHRNA4*-iso2-uORF2; 5, *CHRNA4*-iso2-uORF3; 6, *CHRNA4*-iso2-uORF4; 7, *CHRNA4*-iso2-uORF5. Error bars represent ± SEM of 3 biological replicates. Asterisks indicate significant differences (*p*<0.05).

Two different *CHRNA4* mRNA variants exist. Isoform 1 (NM_000744.6) exhibits a 5′UTR length of 231 nt whereas isoform 2 (NM_001256573.1) is characterized by a longer 5′UTR (690 nt) due to coding region differences which result in a further downstream translation initiation. In the 5′UTR of *CHRNA4* isoform 1 one putative uORF of 60 nt length could be located *in silico* (Fig. 1). Its start codon is encompassed by an adequate Kozak consensus sequence (because of the guanine at position -3). The constructs *CHRNA4*-iso1-1 and *CHRNA4*-iso1-2 were tested by luciferase assay (Table 1 and S1). The results revealed that switching off the uORF significantly increased *CHRNA4*-iso1-1 protein expression by 4.8 fold (*CHRNA4*-iso1-1 0.6±0.09; *CHRNA4*-iso1-2 2.9±0.43; *p* = 0.013) (Fig. 2A).

Some but not all uORFs are translated into protein. In a next step we therefore examined if the ATG start codon of the *CHRNA4-*iso1-1 uORF is able to initiate protein translation, which would be a prerequisite for the translation of the uORF itself. A translated uORF with a mutated stop codon, cloned in frame with the firefly ORF should lead to the synthesis of an elongated firefly protein. The two constructs *CHRNA4-*iso1-3 and *CHRNA4-*iso1-4 were compared to *CHRNA4*-iso1-2. The uORF was set in frame with the firefly ORF for all 3 constructs. The results showed neither a significant difference in protein expression changes between *CHRNA4*-iso1-2 and *CHRNA4-*iso1-3 nor between *CHRNA4*-iso1-2 and *CHRNA4-*iso1-4 (*CHRNA4*-iso1-2 1.47±0.59; *CHRNA4*-iso1-3 0.94±0.25; *p* = 0.542; *CHRNA4*-iso1-2 1.47±0.59; *CHRNA4*-iso1-4 0.83±0.26; *p* = 0.471). Comparison of *CHRNA4*-iso1-4 with the control vector pGL4.10 revealed an increase in protein expression that did not reach significance (*CHRNA4*-iso1-4 0.83±0.26; pGL4.10 0.03±0.01; *p* = 0.067) (Fig. 2B).

To analyse if the results of the luciferase assay were due to a transcriptional or a translational effect, we performed relative quantitative real-time PCR (qPCR). When comparing the mRNA amount of the cells transfected with the constructs *CHRNA4*-iso1-1 and *CHRNA4*-iso1-2 no significant changes were observed between intact and deleted uORF (*CHRNA4*-iso1-1 1.23±0.29; *CHRNA4*-iso1-2 1.05±0.22; *p* = 0.702) (Fig. 2C).

The 5′UTR of *CHRNA4* isoform 2 contains five putative uORFs ranging from 18 to 60 nt in length (Fig. 1). The construct with only intact uORFs was compared to constructs in which each one of the five uORFs was switched off (Table 1 and S1). The results showed no significant differences concerning intact versus switched-off uORF. This is particularly true for the most 5′ uORF which is present in both *CHRNA4* isoforms. This uORF appeared to be functional in the isoform with the shorter 5′UTR (see above) but caused no changes in protein expression when switched off in the isoform with the longer 5′UTR (Fig. 2D). (*CHRNA4*-iso2-uORF1-5 0.12±0.02; *CHRNA4*-iso2-uORF1 0.13±0.02; *p* = 0.644; *CHRNA4*-iso2-uORF1-5 0.12±0.02; *CHRNA4*-iso2-uORF2 0.14±0.03; *p* = 0.607; *CHRNA4*-iso2-uORF1-5 0.12±0.02; *CHRNA4*-iso2-uORF3 0.14±0.02; *p* = 0.427; *CHRNA4*-iso2-uORF1-5 0.12±0.02; *CHRNA4*-iso2-uORF4 0.15±0.03; *p* = 0.514; *CHRNA4*-iso2-uORF1-5 0.12±0.02; *CHRNA4*-iso2-uORF5 0.12±0.02; *p* = 0.926).

When comparing the two wildtype isoforms *CHRNA4*-iso1-1 and *CHRNA4*-iso2-uORF1-5, the results showed that the protein expression of the latter one was significantly reduced (*CHRNA4*-iso1-1 0.6±0.09; *CHRNA4*-iso2-uORF1-5 0.12±0.02; *p* = 0.014).

### *CHRNA5* 5′UTR contains a post-transcriptionally functional uORF

*CHRNA5* 5′UTR (NM_000745.3) was found to harbour one putative uORF of 30 nt length (Fig. 1). Within this uORF the SNP rs56182392 with the ancestral allele guanine (allele frequency 99.4%) and the rare allele adenine results in an amino acid exchange from alanine to threonine when translated.

**Figure 3:**
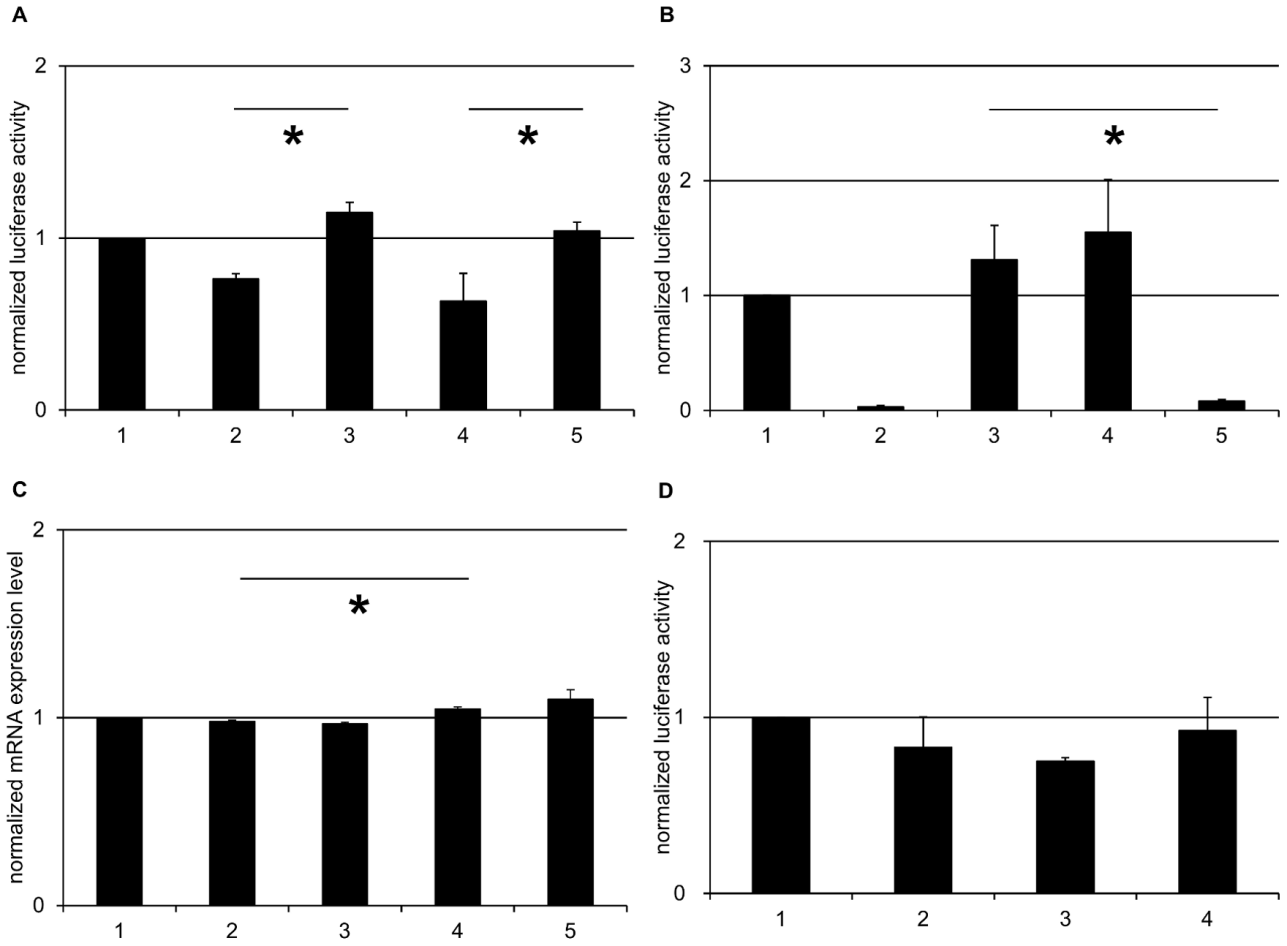
***CHRNA5*** **5** ′**UTR harbours a functional uORF;** ***CHRNB3*** **uORFs are not involved in translational control.** **A:** Luciferase assay of *CHRNA5* 5′UTR resulted in significant increase in protein expression when switching off the uORF. Fold change of firefly activity normalized to pGl4.10 +TK, is illustrated for the five different constructs; 1, pGl4.10+TK; 2, *CHRNA5*-1; 3, *CHRNA5*-2; 4, *CHRNA5*-3; 5, *CHRNA5*-4. **B:** The uORF start codon of *CHRNA5* did not initiate translation as efficiently as the firefly start codon. Fold change of firefly activity normalized to pGl4.10 +TK, is illustrated for the five different constructs; 1, pGl4.10+TK; 2, pGL4.10; 3, *CHRNA5*-2; 4, *CHRNA5*-5; 5, *CHRNA5*-6. **C:** Relative qPCR for *CHRNA5* 5′UTR showed no significant differences of mRNA amount when comparing intact with deleted uORF. However, significant differences were assessed for the transcription of the two SNP allele variants. Fold change of firefly mRNA normalized to pGl4.10 +TK, is illustrated for the five different constructs; 1, pGl4.10+TK; 2, *CHRNA5*-1; 3, *CHRNA5*-2; 4, *CHRNA5*-3; 5, *CHRNA5*-4. **D:** Luciferase assay of *CHRNB3* 5′UTR showed no significant results. Fold change of firefly activity normalized to pGl4.10 +TK, is illustrated for the four different constructs; 1, pGl4.10+TK; 2, *CHRNB3*-1; 3, *CHRNB3*-2; 4, *CHRNB3*-3. Error bars represent ± SEM of 3 biological replicates. Asterisks indicate significant differences (*p*<0.05).

To analyse the *CHRNA5* uORF and its SNP on protein level we tested our constructs by luciferase assays (Table 1 and S1). The constructs *CHRNA5*-2 and *CHRNA5*-4 resulted in a protein increase of 50%, respectively 65%, compared to *CHRNA5*-1, respectively *CHRNA5*-3 (*CHRNA5*-1 0.76±0.03; *CHRNA5*-2 1.15±0.06; *p* = 0.009; *CHRNA5*-3 0.63±0.08; *CHRNA5*-4 1.04±0.03; *p* = 0.006). Consequently, in both cases the constructs lacking the uORF produced significantly more protein than the corresponding constructs with intact uORFs. When comparing the two alleles of rs56182392 (constructs *CHRNA5*-1 and *CHRNA5*-3) the registered protein changes were not significant (Fig. 3A).

Again, to evaluate if the ATG start codon of the *CHRNA5* uORF is able to initiate protein translation, the two constructs *CHRNA5*-5 and *CHRNA5*-6 were compared to *CHRNA5*-2. The uORF was set in frame with the firefly ORF for all 3 constructs. The results revealed that as long as the firefly start codon was intact the luciferase protein was highly expressed (*CHRNA5*-2 1.31±0.30; *CHRNA5*-5 1.55±0.46; *p* = 0.736). However, protein expression dropped by more than 90% when solely the ATG of the uORF was present (*CHRNA5*-2 1.31±0.30; *CHRNA5*-6 0.08±0.01; *p* = 0.029). Comparison of *CHRNA5*-6 with the control vector pGL4.10 showed no significant differences (*CHRNA5*-6 0.08±0.01; pGL4.10 0.03±0.01; *p* = 0.069) (Fig. 3B).

We then performed relative qPCR to rule out an effect on RNA level as a cause for our findings. When comparing the mRNA amount of the cells transfected with the respective constructs (*CHRNA5*-1; *CHRNA5*-2; *CHRNA5*-3; *CHRNA5*-4) by relative qPCR no significant changes were observed between intact and deleted uORF (*CHRNA5*-1 0.98±0.01; *CHRNA5*-2 0.97±0.01; *p* = 0.369; *CHRNA5*-3 1.05±0.01; *CHRNA5*-4 1.10±0.05; *p* = 0.459) (Fig. 3C). Thus, the uORF did not quantitatively alter the mRNA level. Construct *CHRNA5*-3 containing the rare A allele of rs56182392 yielded significantly more firefly luciferase transcripts than *CHRNA5*-1 (*CHRNA5*-1 0.98±0.01; *CHRNA5*-3 1.05±0.01; *p* = 0.011). Nevertheless, no significant differences between the two alleles were seen on protein level and it is therefore unlikely that rs56182392 has an important impact on *CHRNA5* function.

### *CHRNB3* uORF does not influence protein expression

The 5′UTR of this subunit (NM_000749.3) contains a single putative uORF. It harbours a SNP (rs4950 adenine/guanine) with the minor allele guanine deleting the first atg start codon of the putative uORF, creating a truncated, putative uORF (Fig. 1). The three constructs (*CHRNB3*-1; *CHRNB3*-2; *CHRNB3*-3) were compared to each other by luciferase assay (Table 1 and S1). No significant differences were observed between the three different variants (*CHRNB3*-1 0.83±0.17; *CHRNB3*-2 0.75±0.02; *p* = 0.495; *CHRNB3*-1 0.83±0.17; *CHRNB3*-3 0.92±0.19; *p* = 0.778; *CHRNB3*-2 0.75±0.02; *CHRNB3*-3 0.92±0.19; *p* = 0.726). Thus, our findings indicate that neither the longer uORF nor the truncated uORF version play a role in the regulation of protein translation (Fig. 3D).

## Discussion

The data presented here strongly suggest that uORFs within the 5′UTR of *CHRNA4*-iso1 and *CHRNA5* are important regulators of protein translation. Furthermore, they indicate that the two known *CHRNA4* isoforms differ with respect to their effect on gene expression level. The influence of *CHRNA4*-UTR on gene expression was also previously reported by Briggs et al. They expressed ferret *CHRNA4* and *CHRNB2* together with/or without their corresponding UTRs in oocytes. Interestingly, exclusively the high-sensitivity and no low-sensitivity receptor type was detected when the UTR was present. However, putative regulatory UTR elements were not analysed in detail in this study [23].

Compared to the shorter isoform, the presence of the long 5′UTR of *CHRNA4* significantly reduced luciferase expression. None of the five uORFs present in this isoform showed any effect on gene expression when knocked out individually. This renders it unlikely that they are responsible for reduced gene expression levels associated with the longer *CHRNA4* isoform. We cannot completely exclude the possibility that a simultaneous knock out of all five uORFs would have had an effect on gene expression, however, the presence of other repressive regulatory sequence motifs or RNA folding events that either destabilize the mRNA or slow down translation seem to be at least as likely explanations.

Interestingly, the uORF found to be functional in the *CHRNA4* isoform with the shorter 5′UTR is also present in the long 5′UTR-isoform but does not appear to reduce gene expression in the latter sequence context. To our knowledge this presents the first example of a uORF with isoform-specific functional relevance. The reasons behind this observation are so far unknown. Our elongation experiments suggest that the ATG of the short isoform is able to initiate translation of the uORF. However, this effect was rather small and the results were not significant. Therefore translation of the uORF protein might contribute but is unlikely to be the main cause for this observation. It is also possible that the adequate Kozak consensus sequence [24] encompassing the uORF start codon enables sufficient ribosome recognition. Ribosome stalling has therefore to be considered as an alternative mechanism for the effects observed at protein level. It can only be speculated why the same uORF appeared to be non-functional when analysed in the long 5′UTR-*CHRNA4* isoform. A possible reason could be the presence of additional, so far unknown regulatory motifs that only exist in the long 5′UTR-*CHRNA4* 5′UTR and are able to inhibit uORF function. It has already been shown that the regulatory role of at least some uORFs can be closely linked to other 5′UTR sequence motifs [25]. However, the uORF described in this previous report was only functional if additional regulatory motifs were present. The *CHRNA4* uORF discussed here would require the opposite mechanism, i.e. a repression by sequence motifs unknown.

Concerning the single uORF of *CHRNA5*, our experiments showed no clear indication that the uORF start codon is able to initiate translation of the firefly gene cloned downstream of it. The most likely explanation for this observation would be the low-strength Kozak sequence surrounding the uORF-ATG. This weak sequence motif is likely to reduce the probability that ribosomes recognize the uORF-ATG and initiate translation at this site. However, the fact that we were not able to observe a significant difference in expression levels between the control vector and a construct in which the uORF start codon served as the only functioning translation initiation site (*CHRNA5*-6) does not necessarily implicate that translation of the uORF is completely excluded as a possible mechanism. Yet, given the impressive effect on protein expression the uORF showed in the presence of its own intact stop codon it is unlikely that this strong effect resulted solely from a weak translation of the uORF protein. This suggests that, unlike the *CHRNA4* uORF described above, the *CHRNA5* uORF is not part of the group of sequence-dependent uORFs which achieve their effects by encoding small proteins that play active roles in translational control mechanisms. Thus other mechanisms have to be taken into consideration, including stalling of ribosomes at the uORF or leaky scanning. The stalling mechanism occurs when ribosomes stop moving during uORF translation [26]. However, a start codon that is unable to achieve sufficient translation is also unlikely to cause significant ribosome stalling. Leaky scanning seems to be a more plausible explanation for the observed effects. This mechanism particularly occurs if the start codon of the uORF is encompassed by a poor start codon context sequence as present in the uORF of *CHRNA5*. As a result, some ribosomes recognize the start codon of the uORF and initiate, others bypass it and initiate at the next start codon. Because part of the ribosomes and involved translation factors are bound to the uORF, fewer are available for the translation of the main ORF [24], [27]. In general, it has to be borne in mind that although reporter gene assays are an established method they are performed with recombinant proteins and might therefore not completely reflect the *in vivo* situation. In conclusion, uORFs in two of the nAChRs associated with different aspects of nicotine dependence and disorders caused by smoking were found to be functionally relevant. It will be interesting to find out how these regulatory mechanisms contribute to the roles *CHRNA4* and *CHRNA5* have in the pathogenesis of nicotine-related clinical phenotypes.

## Supporting Information

Figure S1 **Standard curves of the qPCR primers with a dilution factor of 10 and duplicate technical samples.** **A:** primer pair firefly-fwd and firefly-rev; E: 97.0%; Rˆ2: 0.985; Slope: −3.40 **B:** primer pair renilla-fwd and renilla-rev; E: 93.5%; Rˆ2. 0.994; Slope: −3.49 E, amplification efficiency; Rˆ2, coefficient of determination
(TIF)

Table S1 **Sequence, terms and restriction sites of the used constructs.**
(DOC)
