# Supplementary figures and images for: Nicotinic Acetylcholine Receptor Subunits α4 and α5 Associated with Smoking Behaviour and Lung Cancer Are Regulated by Upstream Open Reading Frames
Source: PLoS One. 2013 Jul 2;8(7):e66157. doi: 10.1371/journal.pone.0066157 (PMC3699600; doi:10.1371/journal.pone.0066157)

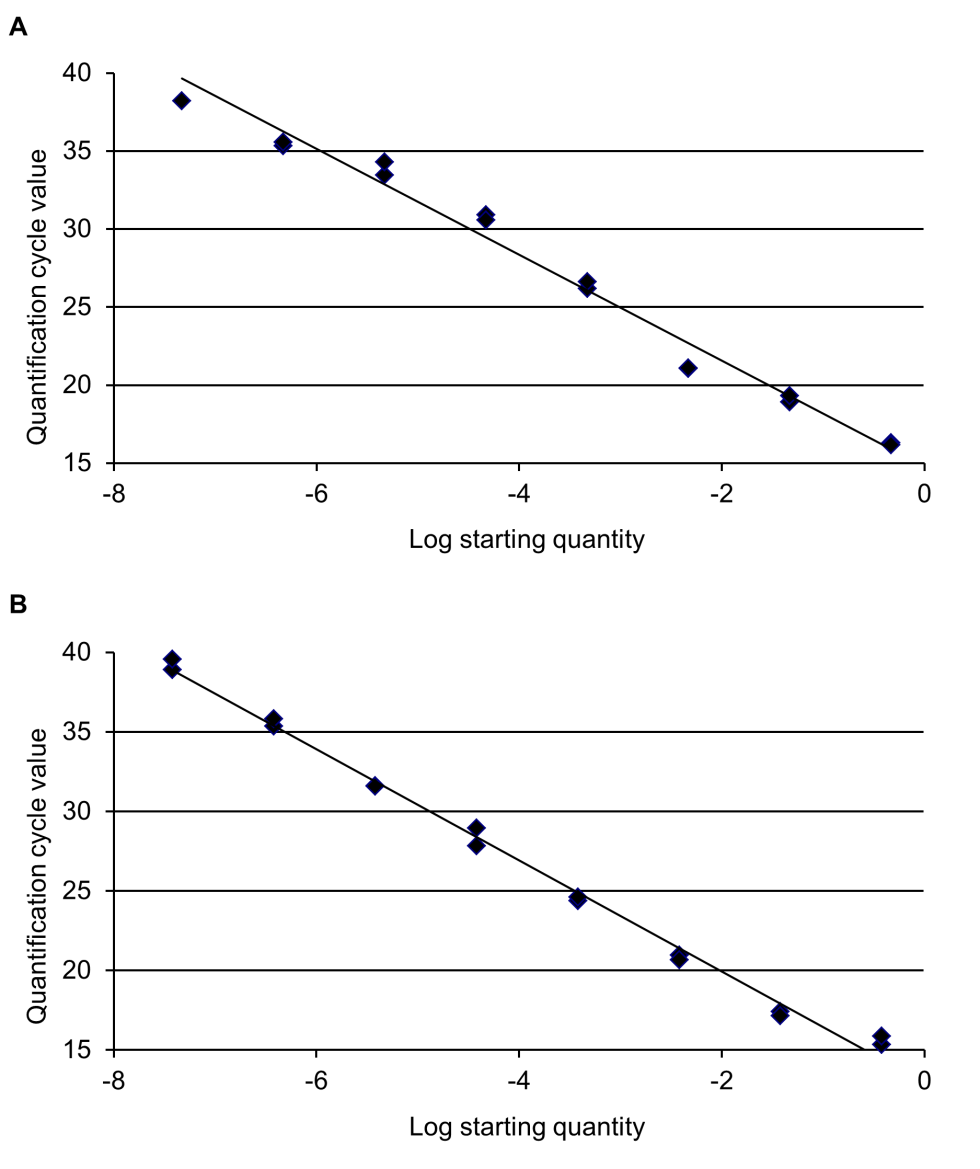

Supplement: Figure S1 — Standard curves of the qPCR primers with a dilution factor of 10 and duplicate technical samples. A: primer pair firefly-fwd and firefly-rev; E: 97.0%; Rˆ2: 0.985; Slope: −3.40 B: primer pair renilla-fwd and renilla-rev; E: 93.5%; Rˆ2. 0.994; Slope: −3.49 E, amplification efficiency; Rˆ2, coefficient of determination (TIF) [file pone.0066157.s001.tif]
