# Supplementary material for: Nicotinic Acetylcholine Receptor Subunits α4 and α5 Associated with Smoking Behaviour and Lung Cancer Are Regulated by Upstream Open Reading Frames
Source: PLoS One. 2013 Jul 2;8(7):e66157. doi: 10.1371/journal.pone.0066157 (PMC3699600; doi:10.1371/journal.pone.0066157)
Supplement: Table S1 — Sequence, terms and restriction sites of the used constructs. (DOC) [file pone.0066157.s002.doc]

**Table S1. Sequence, terms and restriction sites of the used constructs.**

| **Subunit** | **NM_Number** | **Name** | **Sequence with XhoI and NcoI cutting site (italic) and uORF (bold)** |
| --- | --- | --- | --- |
| *CHRNA4* isoform 1 | 000744.6 | *CHRNA4*-iso1-1 | *ctcgag*tcccagccggctgaggcgggcagggccgggcggggccgcgccacggagcccacagcccggcgctccctgccgcgccgccgccgcaccgcgccccacaggagaagacgaaccgggcccggcggccgaagcggcccgcgaggcgcgggaggc**atg**aagttgggcgcgcacgggcctcgaagcggcggggagccgggagccgcccgcatc**tag**agcccgcgaggtgcgtgcg*ccatgg* |
|  |  | *CHRNA4*-iso1-2 | *ctcgag*tcccagccggctgaggcgggcagggccgggcggggccgcgccacggagcccacagcccggcgctccctgccgcgccgccgccgcaccgcgccccacaggagaagacgaaccgggcccggcggccgaagcggcccgcgaggcgcgggaggc**ttg**aagttgggcgcgcacgggcctcgaagcggcggggagccgggagccgcccgcatc**tag**agcccgcgaggtgcgtgcg*ccatgg* |
|  |  | *CHRNA4*-iso1-3 | *ctcgag*tcccagccggctgaggcgggcagggccgggcggggccgcgccacggagcccacagcccggcgctccctgccgcgccgccgccgcaccgcgccccacaggagaagacgaaccgggcccggcggccgaagcggcccgcgaggcgcgggaggc**atg**aagttgggcgcgcacgggcctcgaagcggcggggagccgggagccgcccgcatc**tac**agcccgcgaggtgcgtgcg*cc****atg****g* |
|  |  | *CHRNA4*-iso1-4 | *ctcgag*tcccagccggctgaggcgggcagggccgggcggggccgcgccacggagcccacagcccggcgctccctgccgcgccgccgccgcaccgcgccccacaggagaagacgaaccgggcccggcggccgaagcggcccgcgaggcgcgggaggc**ttg**aagttgggcgcgcacgggcctcgaagcggcggggagccgggagccgcccgcatc**tac**agcccgcgaggtgcgtgcg*cc****ttg****g* |
| *CHRNA4* isoform 2 | 001256573.1 | *CHRNA4*-iso2-uORF1-5 | *ctcgag*tcccagccggctgaggcgggcagggccgggcggggccgcgccacggagcccacagcccggcgctccctgccgcgccgccgccgcaccgcgccccacaggagaagacgaaccgggcccggcggccgaagcggcccgcgaggcgcgggaggc**atg**aagttgggcgcgcacgggcctcgaagcggcggggagccgggagccgcccgcatc**tag**agcccgcgagccagcagccatgtggagacccgggcccacgccgaggagcggctcctgaagaaactcttctccggttacaacaagtggtcccgacccgtggccaacatctcggacgtggtcctcgtccgcttcggcctgtccatcgctcagctcattgacgtggatgagaagaaccagatgatgaccacgaacgtatgggtgaagcaggagtggcacgactacaagctgcgctgggacccagctgactatgagaatgtcacctccatccgcatcccctccgagctcatctggcggccggacatcgtcctctacaacaagtgagtcctctacaacaatgctgacggggacttcgcggtcacccacctgaccaaggcccacctgttccatgacgggcgggtgcagtggactcccccggcatttacaagagctcctgcagcatcgacgtcaccttcttccccttcgaccagcagaactgca*ccatgg* |
|  |  | *CHRNA4*-iso2-uORF1 | *ctcgag*tcccagccggctgaggcgggcagggccgggcggggccgcgccacggagcccacagcccggcgctccctgccgcgccgccgccgcaccgcgccccacaggagaagacgaaccgggcccggcggccgaagcggcccgcgaggcgcgggaggc**ttg**aagttgggcgcgcacgggcctcgaagcggcggggagccgggagccgcccgcatc**tag**agcccgcgagccagcagccatgtggagacccgggcccacgccgaggagcggctcctgaagaaactcttctccggttacaacaagtggtcccgacccgtggccaacatctcggacgtggtcctcgtccgcttcggcctgtccatcgctcagctcattgacgtggatgagaagaaccagatgatgaccacgaacgtatgggtgaagcaggagtggcacgactacaagctgcgctgggacccagctgactatgagaatgtcacctccatccgcatcccctccgagctcatctggcggccggacatcgtcctctacaacaagtgagtcctctacaacaatgctgacggggacttcgcggtcacccacctgaccaaggcccacctgttccatgacgggcgggtgcagtggactcccccggcatttacaagagctcctgcagcatcgacgtcaccttcttccccttcgaccagcagaactgca*ccatgg* |
|  |  | *CHRNA4*-iso2-uORF2 | *ctcgag*tcccagccggctgaggcgggcagggccgggcggggccgcgccacggagcccacagcccggcgctccctgccgcgccgccgccgcaccgcgccccacaggagaagacgaaccgggcccggcggccgaagcggcccgcgaggcgcgggaggcatgaagttgggcgcgcacgggcctcgaagcggcggggagccgggagccgcccgcatctagagcccgcgagccagcagcc**ttg**tggagacccgggcccacgccgaggagcggctcc**tga**agaaactcttctccggttacaacaagtggtcccgacccgtggccaacatctcggacgtggtcctcgtccgcttcggcctgtccatcgctcagctcattgacgtggatgagaagaaccagatgatgaccacgaacgtatgggtgaagcaggagtggcacgactacaagctgcgctgggacccagctgactatgagaatgtcacctccatccgcatcccctccgagctcatctggcggccggacatcgtcctctacaacaagtgagtcctctacaacaatgctgacggggacttcgcggtcacccacctgaccaaggcccacctgttccatgacgggcgggtgcagtggactcccccggcatttacaagagctcctgcagcatcgacgtcaccttcttccccttcgaccagcagaactgca*ccatgg* |
|  |  | *CHRNA4*-iso2-uORF3 | *ctcgag*tcccagccggctgaggcgggcagggccgggcggggccgcgccacggagcccacagcccggcgctccctgccgcgccgccgccgcaccgcgccccacaggagaagacgaaccgggcccggcggccgaagcggcccgcgaggcgcgggaggcatgaagttgggcgcgcacgggcctcgaagcggcggggagccgggagccgcccgcatctagagcccgcgagccagcagccatgtggagacccgggcccacgccgaggagcggctcctgaagaaactcttctccggttacaacaagtggtcccgacccgtggccaacatctcggacgtggtcctcgtccgcttcggcctgtccatcgctcagctcattgacgtgg**ttg**agaagaaccaga**tga**tgaccacgaacgtatgggtgaagcaggagtggcacgactacaagctgcgctgggacccagctgactatgagaatgtcacctccatccgcatcccctccgagctcatctggcggccggacatcgtcctctacaacaagtgagtcctctacaacaatgctgacggggacttcgcggtcacccacctgaccaaggcccacctgttccatgacgggcgggtgcagtggactcccccggcatttacaagagctcctgcagcatcgacgtcaccttcttccccttcgaccagcagaactgca*ccatgg* |
|  |  | *CHRNA4*-iso2-uORF4 | *ctcgag*tcccagccggctgaggcgggcagggccgggcggggccgcgccacggagcccacagcccggcgctccctgccgcgccgccgccgcaccgcgccccacaggagaagacgaaccgggcccggcggccgaagcggcccgcgaggcgcgggaggcatgaagttgggcgcgcacgggcctcgaagcggcggggagccgggagccgcccgcatctagagcccgcgagccagcagccatgtggagacccgggcccacgccgaggagcggctcctgaagaaactcttctccggttacaacaagtggtcccgacccgtggccaacatctcggacgtggtcctcgtccgcttcggcctgtccatcgctcagctcattgacgtggatgagaagaaccagatgatgaccacgaacgt**ttg**ggtgaagcaggagtggcacgactacaagctgcgctgggacccagc**tga**ctatgagaatgtcacctccatccgcatcccctccgagctcatctggcggccggacatcgtcctctacaacaagtgagtcctctacaacaatgctgacggggacttcgcggtcacccacctgaccaaggcccacctgttccatgacgggcgggtgcagtggactcccccggcatttacaagagctcctgcagcatcgacgtcaccttcttccccttcgaccagcagaactgca*ccatgg* |
|  |  | *CHRNA4*-iso2-uORF5 | *ctcgag*tcccagccggctgaggcgggcagggccgggcggggccgcgccacggagcccacagcccggcgctccctgccgcgccgccgccgcaccgcgccccacaggagaagacgaaccgggcccggcggccgaagcggcccgcgaggcgcgggaggcatgaagttgggcgcgcacgggcctcgaagcggcggggagccgggagccgcccgcatctagagcccgcgagccagcagccatgtggagacccgggcccacgccgaggagcggctcctgaagaaactcttctccggttacaacaagtggtcccgacccgtggccaacatctcggacgtggtcctcgtccgcttcggcctgtccatcgctcagctcattgacgtggatgagaagaaccagatgatgaccacgaacgtatgggtgaagcaggagtggcacgactacaagctgcgctgggacccagctgactatgagaatgtcacctccatccgcatcccctccgagctcatctggcggccggacatcgtcctctacaacaagtgagtcctctacaaca**ttg**ctgacggggacttcgcggtcacccacc**tga**ccaaggcccacctgttccatgacgggcgggtgcagtggactcccccggcatttacaagagctcctgcagcatcgacgtcaccttcttccccttcgaccagcagaactgca*ccatgg* |
| *CHRNA5* | 000745.3 | *CHRNA5*-1 | *ctcgag*cttccac**atg**cgtcccgagccc**g**ccagaagctgc**tag**gctgaggctgctgtcccggcgggagctgtggcgcggagcggcccctctgctgcgtctgccctcgttttgtctcacgactcacactcagtgctccattccccaagagttcgcgttccccgcgcggcggtcgagaggcggctgcccgcggtcccgcgcgggcgcgggg*ccatgg* |
|  |  | *CHRNA5*-2 | *ctcgag*cttccac**ttg**cgtcccgagccc**g**ccagaagctgc**tag**gctgaggctgctgtcccggcgggagctgtggcgcggagcggcccctctgctgcgtctgccctcgttttgtctcacgactcacactcagtgctccattccccaagagttcgcgttccccgcgcggcggtcgagaggcggctgcccgcggtcccgcgcgggcgcgggg*ccatgg* |
|  |  | *CHRNA5*-3 | *ctcgag*cttccac**atg**cgtcccgagccc**a**ccagaagctgc**tag**gctgaggctgctgtcccggcgggagctgtggcgcggagcggcccctctgctgcgtctgccctcgttttgtctcacgactcacactcagtgctccattccccaagagttcgcgttccccgcgcggcggtcgagaggcggctgcccgcggtcccgcgcgggcgcgggg*ccatgg* |
|  |  | *CHRNA5*-4 | *ctcgag*cttccac**ttg**cgtcccgagccc**a**ccagaagctgc**tag**gctgaggctgctgtcccggcgggagctgtggcgcggagcggcccctctgctgcgtctgccctcgttttgtctcacgactcacactcagtgctccattccccaagagttcgcgttccccgcgcggcggtcgagaggcggctgcccgcggtcccgcgcgggcgcgggg*ccatgg* |
|  |  | *CHRNA5*-5 | *ctcgag*cttccac**atg**cgtcccgagccc**g**ccagaagctgc**tac**gctgaggctgctgtcccggcgggagctgtggcgcggagcggcccctctgctgcgtctgccctcgttttgtctcacgactcacactcagtgctccattccccaagagttcgcgttccccgcgcggcggtcgagaggcggctgcccgcggtcccgcgcgggcgcgggg*cc****atg****g* |
|  |  | *CHRNA5-*6 | *ctcgag*cttccac**atg**cgtcccgagccc**g**ccagaagctgc**tac**gctgaggctgctgtcccggcgggagctgtggcgcggagcggcccctctgctgcgtctgccctcgttttgtctcacgactcacactcagtgctccattccccaagagttcgcgttccccgcgcggcggtcgagaggcggctgcccgcggtcccgcgcgggcgcgggg*cc****ttg****g* |
| *CHRNB3* | 000749.3 | *CHRNB3*-1 | *ctcgag*tgttgctgtcctcttgggttccacttcggattttgaacccctgtattttcttttcaaaacccccttttcca**atg**gaaatgctctgttgt**taa**aaaggaagaaactgtctttctgaaactgacatca*ccatgg* |
|  |  | *CHRNB3*-2 | *ctcgag*tgttgctgtcctcttgggttccacttcggattttgaacccctgtattttcttttcaaaacccccttttcca**gtg**gaa**atg**ctctgttgt**taa**aaaggaagaaactgtctttctgaaactgacatca*ccatgg* |
|  |  | *CHRNB3*-3 | *ctcgag*tgttgctgtcctcttgggttccacttcggattttgaacccctgtattttcttttcaaaacccccttttcca**gtg**gaa**ttg**ctctgttgt**taa**aaaggaagaaactgtctttctgaaactgacatca*ccatgg* |
